# Costs, outcomes and challenges for diabetes care in Spain

**DOI:** 10.1186/1744-8603-9-17

**Published:** 2013-05-01

**Authors:** Julio Lopez-Bastida, Mauro Boronat, Juan Oliva Moreno, Willemien Schurer

**Affiliations:** 1 University Castilla La Mancha, Avda Real Fábrica de Seda s/n, Talavera de la Reina, Toledo 45600, Spain; 2 Red de Investigación en Servicios Sanitarios en Enfermedades Crónicas (REDISSEC), Spain; 3 Section of Endocrinology and Nutrition, Hospital Universitario Insular, Avda. Marítima del Sur, s/n, Las Palmas de Gran Canaria 35016, Spain; 4 University Castilla La Mancha, Cobertizo de San Pedro Mártir s/n, Toledo 45071, Spain; 5 LSE Health, London School of Economics, Houghton Street, London WC2A 2AE, UK

**Keywords:** Diabetes, Costs, Outcomes, Quality of care, Prevalence, Incidence, Spain

## Abstract

**Background:**

Diabetes is becoming of increasing concern in Spain due to rising incidence and prevalence, although little information is known with regards to costs and outcomes. The information on cost of diabetes in Spain is fragmented and outdated. Our objective is to update diabetes costs, and to identify outcomes and quality of care of diabetes in Spain.

**Methods:**

We performed systematic searches from secondary sources, including scientific literature and government data and reports.

**Results:**

Diabetes Type II prevalence is estimated at 7.8%, and an additional 6% of the population is estimated to be undiagnosed. Four Spanish diabetes cost studies were analyzed to create a projection of direct costs in the NHS and productivity losses, estimating €5.1 billion for direct costs along with €1.5 billion for diabetes-related complications (2009) and labour productivity losses represented €2.8 billion. Glycemic control (glycolysated hemoglobin) is considered acceptable in 59% of adult Type II cases, in addition to 85% with HDL cholesterol ≥40mg/dl and 65% with blood pressure <140/90 mmHg, pointing to good intermediate outcomes. However, annual figures indicate that over half of the Type II diabetics are obese (BMI >30), 15% have diabetic retinopathy, 16% with microalbuminuria, and 15% with cardiovascular disease.

**Conclusions:**

The direct health care costs (8% of the total National Health System expenditure) and the loss of labour productivity are high. The importance of a multi-sectoral approach in prevention and improvements in management of diabetes are discussed, along with policy considerations to help modify the disease course.

## Background

Diabetes mellitus (DM) is a leading health problem globally. DM is one of the leading causes of mortality and major morbidities, including cardiovascular disease (CVD), kidney failure, amputations and blindness [1]. The risk of developing these conditions is higher for diabetic patients compared with non-diabetic patients in the same age-group [2,3].

A longitudinal chronic disease study found that diabetes incidence increased with age, with almost 20% prevalence in people aged over 65 years, and half all diabetic patients are aged over 65 years. By 2050, diabetes cases are projected to increase four-fold in patients older than 70 years [4]. It requires a continuous lifetime medical model to slow down the development of complications, improve quality of life and minimize its high social costs. The development of diabetes-related complications depends on glycemic control, but also on cardiovascular risk factors such as obesity, poor diet and inactivity. Multidisciplinary health care, along with high patient involvement, is integral to good diabetes management.

In Spain, diabetes exerts a great impact on public health due to its high prevalence (6.2% aged 30–65 years; 10% aged 30–89 years) [5,6], but also for its acute and chronic complications and high mortality rate. Diabetes Type I prevalence is 0.2-0.3%, while annual incidence under 14 years of age is 9.5-16/100,000 inhabitants annually; and in 15 to 29 years of age is 9.9/100,000 inhabitants annually. In children under 5 years, incidence is minimal. There was no discernible difference in diabetes incidence between the sexes until after the age of 15, when Type I incidence was found to be greater in males [7-10]. The estimated incidence of both Type I and II diabetes is 11-12/100,000 and 8/1000 inhabitants annually, respectively [5,6]. Sole Type II diabetes incidence data is scarce due to insufficient data [11], however, it is accepted that both incidence and prevalence are increasing in Type II with decreasing age at diagnosis.

A national Spanish study based on 5,800 surveys examined diabetes prevalence, obesity and associated risk factors, and found that Type II prevalence was 13.8%, a slight increase over previous studies [1] (Table 1). The study also confirmed the association between diabetes, obesity and hypertension, and the importance of increased physical activity as a preventive measure. While obesity prevalence is almost 30% nationally, there is some concern that 6% of patients with Type II diabetes have not yet been diagnosed of their condition [1].

**Table 1:** Diabetes prevalence in Spain

	**Prevalence %**	**Prevalence Number**
	**(≥18 years)**	**(≥18 years)**
Diabetes: Diagnosed	**7.8%**	**2996395**
Diabetes: Undiagnosed	**6.0 %**	**2304919**
Diabetes: Total	**13.8 %**	**5301314**
Abnormal glucose tolerance	**9.2%**	**3534210**
Impaired fasting glucose	**3.4%**	**1306121**
Obesity: BMI> 30	**28.2%**	**10863431**

***Source:*** Soriguer F et al., 2012 [1].

Lower estimates of diagnosed Type II prevalence have been reported, including 3.1% in Aragon [6], 6.7% in Catalonia [12], 4.6% in the Basque country [13], 4% in Asturias [14], 7.8% in Murcia [15] and 8% in Valencia [16]. The prevalence of diagnosed and undiagnosed Type II was 6.6% in Andalusia [17], 6.1% in Aragon [6], 7.8% in Galicia [18], 10.3% in Catalonia [12], 9% in Asturias [14], 11.7% en Balearic Islands [19], 12% in Canary Islands [20], and 11% in Murcia [15].

In Spain the National Health System (NHS) is a public health care insurance system with universal coverage of 47 million people. Administered by 17 regions, it is coordinated by the national government and fully financed by the general tax fund. Hospitals and primary health care centres are publicly owned, with some contracting out to private hospitals. Medical doctors are paid by salary. Primary care doctors act as gatekeepers. There are private health insurance companies and private practices, where specialists in particular play a complementary role, although some activities are totally private (dentistry). As a percentage of GDP, total health expenditure in Spain is 9.5% in the year 2009 (71% public and 29% private). Public health expenditure represents 7.0% of GDP and per capita spending is €1,604 [21].

With regard to diabetes care, Spain offers a good health coverage system with well- developed care free at point of delivery. All diabetes expenses are fully covered by the NHS, with no out of pocket payments. Since 2007, there has been a national diabetes plan providing general guidelines to stimulate the implementation of regional programs for prevention, early diagnosis and efficient treatment, as well as research [22]. Some regions in turn have their own regional prevention plans.

The objectives of this paper are to identify and update diabetes costs, to present outcomes and quality of care, and to discuss challenges raised by diabetes for Spain’s future. Evidence on direct costs and productivity losses of diabetes and related complications over the last decade will be presented as well as making expenditure projections for the Spanish NHS.

### Methods

We performed systematic searches in electronic databases (Medline), grey literature, reports from national and international sources, including professional bodies and organizations, of published papers on the costs and outcomes of diabetes in Spain, period 2000–2010. It was conducted during September and December 2010. In undertaking the research, the following key words were used, both in English and Spanish language: “Spain + diabetes”; “Spain + diabetes + prevalence”; “Spain + diabetes + costs”; “Spain + diabetes + costs + complications”; “Spain + diabetes + outcomes”; “Spain + diabetes + guidelines”. A number of studies were then selected on the basis of their correspondence with the costs, outcomes and organizations pertinent to Spain.

## Results and discussion

The search generated 273 possible references. After reading abstracts or full articles, we identified four major studies [23-26] which investigated the costs of diabetes in Spain, including costs of complications.

The authors selected two national studies (bottom-up and town-down approaches) that had estimated the total cost of diabetes with a health service perspective [23,24]. A top-down regional study that had estimated the cost of diabetes with a broad perspective (societal costs) [25]. Finally, a fourth regional study that while used a bottom-up approach, for its large sample and broad perspective (societal costs) helped to supplement the information contained in the three previous studies [26].

### Cost of diabetes in Spain

All recent Spanish studies on diabetes costs undertaken over the past few years were at national or regional levels. Two national studies [23,24] and two regional studies [25,26], were identified which address direct diabetes costs using a similar approach, which made analysis possible. Therefore making them comparable (Table 2).

**Table 2:** Comparison of annual diabetes costs per patient in Spain

	**Mata et al. (CODE 2)**	**Oliva et al.**	**Ballesta et al.**	**Lopez Bastida et al.**
Publication year	2002	2004	2006	2002
Reference year	1998	2002	1999	1998
Type of study	National, observational, prevalence and bottom-up approach.	National, prevalence and top-down approach.	Regional, observational, prevalence and bottom-up approach.	Regional, prevalence and top-down approach.
Type of costs	Direct health care costs	Direct health care costs	Direct health care costs and productivity losses (human capital)	Direct health care costs and productivity losses (human capital)
Sources of resources used	Primary data.	Primary and secondary data.	Primary data.	Primary data.
Diabetes Type	Type II	Type I & II	Type II	Type I & II
Age	30-89 years	18+ years	14+ years	0-99 years
Total costs	€1305	€1290-1476	€4278	€758
			Direct: €2504	Direct: €470
			Productivity €1774	Productivity: €288
Drug costs	€554	€636	€851	€179
Hospitalization costs	€417	€512	€1005	€183
% inpatient costs	59.8%	37%	39%	38%
% outpatient costs	18.5%	17%	28%	23%
% drug costs	21.7%	46%	33%	39%

***Source:*** Data taken from the cost of illness studies [23-26].

As part of the CODE 2 (Cost of Diabetes in Europe-Type II) study, a national diabetes cost study was undertaken in Spain in 1998 to estimate Type II diabetes direct costs, and to differentiate care costs, costs of complications and other unrelated health costs [24]. This study used a bottom-up approach to costing and a health service perspective. The annual health cost was €1,305/patient of which 28.6% (€373) was for diabetes control, 30.5% (€398) diabetes-related complications, and 40.9% (€534) was unrelated. The mean cost of patients without complications was €883 compared to €1,403 with microvascular and €2,022 with macrovascular complications, and €2,133 with both types of complications [24].

Another national study estimated health care resources spent by Type I and Type II adult diabetic patients in Spain during the year 2002 [23]. This study used a top-down approach and a health service perspective. The estimated 2002 direct costs of diabetic people ranged from €2.4-2.7 billion depending on the prevalence estimate. Hospital costs were highest (€933 million), followed by non-insulin and non-hypoglycemic drugs (€777–932 million). Much lower were the insulin and oral hypoglycemic agents (€311 million), primary care visits (€181–272 million), specialized visits (€127–145 million), and disposable elements (€70–81 million). Expenditures for all drugs and consumable goods ranged between €1.2-1.3 billion, representing 48–49% of total costs, 15% higher than hospital costs. The direct health care costs of diabetic patients were high (6.3–7.4% of total NHS expenditure). The average annual cost was €1,290 –1,476/patient, while non-diabetic was €865/patient [23].

In a smaller regional study, descriptive costing observations of 517 patients with Type II diabetes were collected [26]. This study used a bottom-up approach and they measured productivity losses (indirect costs) and a broad perspective (societal costs). Total annual health costs of €4,278/patient were estimated (direct €2,504; indirect €1,774). Multiple regression analysis showed an independent association between total costs and obesity, male sex, number of diabetes-related hospitalizations, permanent disability, macrovascular complications, and having both micro- and macrovascular complications. Their findings confirm both the high economic cost of Type II diabetes and the direct relationship between costs and diabetes-related complications (obesity, permanent disability and micro-/macrovascular complications) [26].

An older regional cost of illness study evaluated the economic impact of direct health costs and the productivity losses (indirect costs) caused by diabetes in the Canary Islands in 1998 [25]. This study used a top-down approach and a broad perspective (societal costs). The total annual diabetes costs were €39 million, or €758/diagnosed patient. Total annual diabetes direct costs were €24 million, approximately 2.13% of health expenditure; or the equivalent of €470/diagnosed patient. The largest portion of total diabetes costs (direct costs plus productivity losses) was direct costs at 62%, while productivity losses came to €15 million [25].

This data (Table 2) was then used to create national diabetes expenditure projections. The first projection involves the total direct diabetes costs plus productivity losses. The direct health care cost per patient was taken from Oliva et al. [23] and the productivity losses from Lopez-Bastida et al. [25] and Ballesta et al. [26]. The costs were adjusted to 2009 taking into account annual inflation and population data from INE [27] and diabetes prevalence [1] (Table 3).

**Table 3:** Direct and productivity losses of diabetes projections for 2009

**Spain 18+ years**		**Type II diabetes prevalence (%)**	**Type II diabetes patients (n)**	**Annual Cost per patient (€)**	**Total costs (€ million)**
**Direct costs**					
**2009**	38553614	7.8%	3007182	1660	5119.92
**Productivity losses**					
**2009**	38553614	7.8%	3007182	916	2825.21

***Source:*** Authors’ calculations based on INE population data [27], 2002 annual costs per patient [23] (inflated to 2009 using INE inflation rate), and diabetes prevalence [1].

The second projection calculates the overall diabetes-related complications, by applying per patient costs of diabetes-related complications onto the national prevalence. Annual micro- and macrovascular complication costs per patient [23,24] were applied to diabetes prevalence [1] (Table 4).

**Table 4:** Cost of diabetes complications Type II in 2009

	**Total direct costs (€ millions)**	**Direct cost per patient (€)**	**Components of direct costs (€ million, %)**
Macrovascular complications	€757.74	€2776	Pharmaceuticals €263.69 (34.8%)
			Inpatient €334.16 (44.1%)
			Outpatient€159.88 (21.1%)
Microvascular complications	€579.79	€1928	Pharmaceuticals €241.19 (41.6%)
			Inpatient €189.59 (32.7%)
			Outpatient €149 (25.7%)
Micro- and Macrovascular complications	€542.22	€2930	Pharmaceuticals €208.75 (38.5%)
			Inpatient €217.97 (40.2%)
			Outpatient €115.49 (21.3%)

***Source:*** Authors’ calculations based on diabetes prevalence [1], INE and adjusted diabetes-related complication costs updated from Mata et al., 2002 [24].

### Allocation of resources and outcomes

Type I diabetes patients are in general attended by endocrinologists, paediatric endocrinologists and diabetes nurses, frequently as hospital outpatients. Some tertiary hospitals have developed multidisciplinary diabetes units. Information about quality of care and final outcomes of Type I diabetes in Spain is scant, generally limited to single-centre results [28,29].

Type II diabetes patients are mostly treated in a primary care setting, with varying participation of specialists, depending on available human resources as well as local or regional policies. Interventions aimed at improving the coordination and the delimitation of competences between health care levels are currently being promoted [30].

The Group for the Study of Diabetes in Primary Care of Health (GEDAPS) published their first edition of the “Guidelines for the Treatment of Type 2 Diabetes Mellitus in Primary Care” in 1993, with subsequent editions in 1995, 1998, 2000, 2004, and 2010 [31]. These guidelines are based on agreements made by diabetes health professionals, defining quality of care for Type II diabetic patients primarily based on the Saint Vincent Declaration which was adapted for primary care. Other less known Type II guidelines have been launched, including one endorsed by the Ministry of Health [32] and the Spanish Diabetes Society [33]. Only one set of Type I diabetes treatment guidelines are available and created by the Spanish Society of Endocrinology and Nutrition [34]. However, their dissemination has been rather limited.

Subsequent to GEDAPS guidelines, a GEDAPS Network (RedGEDAPS) was created by diabetologists nationally to promote quality improvements, in part via through the collection of intermediate and final complications outcomes (Table 5). Intermediate outcome indicators are glycosylated hemoglobin (HbA1c), total and high-density (HDL) cholesterol, blood pressure, BMI and urinary albumin. Prevalence of complications include diabetic retinopathy, cardiovascular disease (including stroke, MI), foot ulcerations and amputations, microalbuminuria, and hospital admissions with high blood sugar. These indicators are collected infrequently, to date 1996, 1998, 2000, 2002, and 2007. According to the RedGEDAPS audits, all indicators in Type II diabetes patients have progressively improved from 1996 to 2007 [35].

**Table 5:** Type II Intermediate and final diabetes related complications outcome indicators (1996–2007)

**Intermediate**	**1996**	**1998**	**2000**	**2002**	**2007**	Difference (1996–2007)*
**HbA1c ≤7%**	43.0%	49.2%	56.1%	53.4%	59.0%	16.0 (15–17)
**HbA1c ≥10%**	10.1	6.4	5.3	4.5	4.6	5.5 (4.9–6.1)
**HDL Cholesterol >40mg/dl or >35mg/dl if >65 years**	83.1	77.5	80.0	83.1	84.7	1.6 (1.3–1.9)
**Total Cholesterol <250 mg/dl**	75.0	75.4	83.1	78.0	77.5	2.5 (2.1–2.9)
**BMI <30**	62.2	59.3	62.0	56.8	48.9	13.3 (12.4–14.2)
**Blood presure <140/90mmHg**	59.1	53.6	59.6	63.1	65.1	6.0 (5.3–6.7)
**Active smoking**	15.4	16.5	15.9	14.8	11.1	4.3(3.7–4.9)
**Final**	**1996**	**1998**	**2000**	**2002**	**2007**	**Difference 1996–2007**
**Diabetic retinopathy**	34.3%	25.8%	22.7%	17.0%	15.6%	18.7 (17.6–19.8)*
**Diabetic foot ulcers**	5.7	4.1	6.6	2.5	3.1	2.6 (2.2–3.0)*
**Amputation: lower limb**	2.4	1.2	1.2	0.7	0.5	1.9 (1.5–2.3)*
**Microalbuminuria or proteinuria**	15.0	26.9	37.9	21.4	16.7	1.7 (1.3–2.1)*
**Cardiovascular: Stoke, MI**	21.4	18.8	19.0	18.1	14.5	6.9 (6.2–7.6)*
**Hospital admission with blood glucose >500mg/dl: amputation, ketosis, hypoglycemia and other**	5.5	4.3	4.7	4.8	5.9	0.4 (0.2–0.6)

***Source***: Franch-Nadal F.J. et al. [35].

***Note:*** * all differences 1996–2007 are significant.

Diabetic retinopathy is a major cause of blindness in people under 60 years (20-30%), while blindness prevalence amongst diabetes patients is 5-6%, a risk 6 times higher than the general population. Almost all Type I diabetes patients have degeneration just after 14 years after diagnosis. Diabetic retinopathy is a major concern also in Type II patients with 33% retinopathy, as well as 17% with renal complications, 21% peripheral vascular disease, 10% cerebral vascular disease, 14% coronary disease, and 40% neuropathy. In Type II, at least half of patients have some type of complication at the time of diagnosis [36]. Despite improvements over time, diabetes-related complications continue to be significant (Table 5).

In a Spanish national cross-sectional multicenter study comparing 1,041 Type II patients enrolled in 29 primary care centers to 8,693 subjects from the general population, the HRQOL via the generic questionnaire (EQ-5D) was worse among diabetic subjects (mean EQ-5D index score of 0.71 vs 0.81). Diabetes patients with diabetes-related complications, poor glycemic control and insulin treatment had worse HRQOL than diabetes patients without complications, adequate control or no insulin treatment [37].

A similar study applying the HRQOL to 5,549 Canary Island citizens also found that subjects with diabetes presented a worse HRQOL than non-diabetic age- and sex-matched control subjects [38]. Average EQ-5D index score of diabetic people was 0.69 compared to 0.77 of the remaining sample (median EQ-5D index score was 0.83 among diabetic subjects vs. 0.88 among non-diabetic controls). Diabetes patients also had higher prevalence of moderate and severe problems in the five dimensions of EQ-5D (Mobility, Self-care, Usual activities, Pain/Discomfort, Depression/Anxiety) compared to the non-diabetic sample. These findings were concordant with a previous study of adults with diabetes, in which fair or poor health was two-fold higher in non-diabetic than in diabetic subjects, a finding unchanged over the 1993–2003 decade (71.2 to 70.5%) [39].

A 2006 Catalonia health survey (n=15,926) found a significant, moderately negative relationship between diabetes and HRQOL (via EQ=5D) [40]. Further subgroup analysis revealed diabetic subjects without vascular disease or associated risk factors had a similar HRQOL to the non-diabetic population. In contrast, diabetic individuals with vascular risk factors had significantly lower HRQOL than in non-diabetic subjects.

### Challenges and policy recommendations

Major factors underpinning rising prevalence are an ageing population and rising obesity, the latter which has been steadily increasing primarily due to poor diet and low physical activity [41].

Specifically to Type II diabetes in Spain, the following conditions increase the risk of diabetes diagnosis [42]. Obesity is of significant concern in Spain, despite a climate favourable to exercise and a Mediterranean diet. Approximately one-third of adults are overweight (37%) and 15.4% are obese. Even children are at risk for lifetime of chronic obesity-related conditions, with 9.4% of 2–17 year olds recorded as obese and 19.2% as overweight [43]. Approximately one-third of adults do not engage in any daily physical exercise, placing them at risk for developing excess body adipose [43].

Prevention, especially through promoting healthy life styles, must be further enforced in the policy agenda both at national and regional level. Primary prevention of diabetes is effective in decreasing the incidence of diabetes [22].

Scientific data support the efficacy of lifestyle measures used in Type II diabetes prevention, however, realities of program implementation in the general population pose some difficulties. This leads us to believe that socio-health, educational and fiscal policies are needed to implement lifestyle interventions in at-risk populations [44]. The Ministry of Health, in compliance with World Health Organization (WHO) guidance, advocates an integrated approach combining diabetes prevention, diagnosis and treatment [22].

The 2007 government strategy aims to coordinate tools within the NHS using the guiding principles of solidarity, equity and participation to achieve inequality reduction and the promotion of healthy lifestyles and a high quality of care [22]. The strategy was developed by a multidisciplinary team including scientific societies, patients and caregivers, the Ministries of Health of the Autonomous Communities (AC), and the cities of Ceuta and Melilla [22].

To configure this strategy, a survey by the Institutional Committee of the Diabetes Strategy at the NHS explored four main areas: health policy; organizational strategies; clinical strategies; and information systems and data records. Its 2006 results found diabetes was a priority area for health intervention in 94.3% of the Autonomous Communities (AC). Since then, only 31.6% have a diabetes action plan, with the exception of Navarre and Valencia community with plans in place since 1996. These plans are prepared and edited by the Ministries of Health (MOH), usually led mainly by a MOH directorate or regional Health Service.

The AC play a significant role in diabetes policy, as 63.2% have diabetes advisory councils, composed of management, professional, scientific societies and patient associations. 68.5% of ACs have established standard coordination between primary and specialty care, mostly through agreed protocols, improvement committees, clinical sessions and training activities. In addition, 89.5% of ACs have a diabetes plan in primary care, and 84.2% have agreed protocols in place for monitoring DM in the different levels of care[22]. A further 57.5% have defined specific action plans for preventing complications: there are programs for screening for diabetic retinopathy in 84.2% ACs, 84.2% for diabetic foot, and 89.5% for diabetic nephropathy [22].

There are differences in the distribution of endocrinology resources between ACs, although most (84.2%) are located in hospitals and specialty centers. Some regions such as Catalonia, the Baleares and Melilla are centralized in hospitals alone. 52.6% of ACs have day hospitals and single consultations for the continuing care of diabetic patients in health care[22].

In 80% of ACs, most of the actions that are carried out in relation to primary diabetes prevention are integrated into programs to prevent cardiovascular risk, obesity, education, health nutrition and healthy habits. Early detection activities are implemented in 84.2% of ACs, and 52.6% have some mechanism to detect and record the population at risk [22]. In 94.7% of ACs are diabetes education programs, carried out in 89.5% of the time by primary care teams and 68.4% by specialist care services. In 63.2% of ACs, there are additional resources for diabetes education, from specialty care or primary care or both [22].

The Ministry of Health is encouraging the NAOS Strategy (Strategy for Nutrition, Physical Activity, and Obesity Prevention) to reduce obesity [45]. The PERSEUS Program (Pilot Program Reference School for Health and Exercise Obesity) included as one of the Quality Plan for the NHS, is aimed at preventing obesity in schools through interventions aimed at educators and students. In 2011 Spain had implemented a law requiring all schools to serve healthy foods and banned unhealthy ones from the premises.

The evaluation of diabetes patients within the health system is important in order to understand patient behavior and vision of their own health status, and explore their social context and environment variables to identify cultural, organizational and personnel influencing disease evolution. Patients with Type II diabetes, as experienced users of the health system, can be assessed "from below" the care services that treat them and, therefore, provide relevant and useful information to improve quality treatment and health management system [46].

Diabetes presents several challenges to the NHS and the wider Spanish society. The main challenge is to reduce the rising diabetes prevalence. Its evolution represents a real threat to the health of the population along with affordability of the health care system.

The multidimensional nature of the obesity problem will require a multi-faceted solution. The success of policies depends on a combination of measures that take into account cultural, economic and social aspects. As the increase in obesity rates are the result of cultural, social, economic and technological influences (i.e. sedentary jobs, lower prices of food rich in sugar and fat in comparison with fruit and vegetables, the high opportunity cost of cooking at home, the use of private cars instead of public transport, walking or cycling, and so on), it is difficult to identify cost-effective strategies.

Several limitations of this study must be recognized. First, diabetes prevalence is probably higher than the figures suggest due to significant numbers of undiagnosed diabetes cases. It also should be stressed that the present work does not consider non–health care direct costs or informal care costs faced by patients and society as a whole.

## Conclusions

In this paper, we quantified direct medical costs and productivity losses of diabetic patients for 2009 in Spain, and found patients consumed 8% of total public health expenditures. We have estimated €5.1 billion for direct costs, with €1.5 billion for diabetes-related complications. Labour productivity losses amounted to € 2.8 billion. The annual cost per diabetic patients averaged close to €1,660 for direct costs and €916 for productivity losses, with significant differences between patients with and without micro and macrovascular complications.

Diabetes, particularly including its complications, not only affects the health status of the individual, but also their ability to work during their productive life. Consequently, non-health social costs should be also taken into account when assessing the overall economic burden of diabetes. Since most of the Spanish studies relating to the cost of diabetes take into account only health care costs, it is necessary to include labour productivity losses and formal and informal care costs to obtain a better assessment.

The relative weight of different cost items is similar to the findings of other studies, with inpatient costs the primary consumer [47-49]. Many studies omit non-diabetes drug data, which is significant as polypharmacy is a frequent phenomenon among diabetic patients increasing with patient age, disease duration and the presence of complications. Most diabetic patients require diabetes drugs as well as treatment for co-morbidities and diabetes-related complications, particularly cardiovascular disease. Therefore, quality management of diabetes and chronic complications should be imperative.

Resources should not be allocated according to the costs of diseases but rather where interventions will benefit the most [50]. This study shows that diabetes costs illustrates the real dimension of health problems and reveals its true economic burden, quantifying health care and other resources that could be allocated elsewhere [51]. Working towards diabetes prevention as well as prevention of diabetes-related complications is a real goal, of which Spain has implemented a number of programmes to help towards this point.

Diabetes, alone and with its complications, affects individual health status as well as working abilities and productive life. Productivity losses should be accounted for in assessing the overall economic burden of diabetes. The only Spanish study accounting for productivity loss found it represented 36% of overall expenditure [25]. Since most of the Spanish studies accounted only for direct costs, a more comprehensive assessment which includes productivity losses is necessary. Improved diabetes-related accounting within the NHS, along with regular collection and reporting of diabetes indicators and outcomes is needed.

The HRQOL in diabetics is not necessarily lower than in non-diabetics, provided that cardiovascular disease risk factors are under control and good glycaemic control is present. On the other hand, the presence of cardiovascular disease or its risk factors are associated with a significantly diminished quality of life for diabetic persons. The HRQOL should be included within a diabetes dataset including diabetes-related mortality, incidence and prevalence to aid in developing health policy and planning.

It is therefore essential for the Spanish health system to further boost prevention, both primary and secondary activities, as the main tool to reduce the incidence of Type II diabetes and diabetes-related complications. Improved data collection to monitor incidence, prevalence, expenditure and outcome indicators is needed to ascertain the position of diabetes in the Spanish health care system. Ironing out of regional inequalities is important not only for care access but also care planning and education provision. Ultimately, the key to reducing the impact of diabetes on society is in its prevention, and when this is not possible, then to at the very least, ensure good glycemic control to minimize costly diabetes-related complications.

## Abbreviations

DM: Diabetes mellitus; CVD: Cardiovascular disease; NHS: National Health System; GDP: Gross domestic product; INE: Institute national of statistics; HRQOL: Health-related quality of life; EQ-5D: EuroQol EQ-5D.; WHO: World Health Organization; AC: Autonomous communities; MOH: Ministry of health.

## Competing interests

The authors declare that they have no competing interests.

## Authors’ contributions

JLB conceived and designed the research, acquired the data, analyzed and interpreted the data, handled funding and supervision, drafted the manuscript and critically revised the manuscript for important intellectual content. MB acquired the data, analyzed and interpreted the data, critically revised the manuscript for important intellectual content. JOM conceived and designed the research, analyzed and interpreted the data and drafted the manuscript. WS conceived and designed the research, acquired the data, analyzed and interpreted the data, drafted the manuscript and critically revised the manuscript. All authors read and approved the final manuscript.
